# Editorial: Martial arts, health, and society

**DOI:** 10.3389/fsoc.2022.1032141

**Published:** 2022-09-27

**Authors:** George Jennings, Lorenzo Pedrini, Xiujie Ma

**Affiliations:** ^1^Cardiff School of Sport and Health Sciences, Cardiff Metropolitan University, Cardiff, United Kingdom; ^2^Department of Sociology, University of Milano-Bicocca, Milan, Italy; ^3^Chinese Guoshu Academy/The School of Martial Arts at Chengdu Sport University, Chengdu, China

**Keywords:** martial arts, combat sports, health, wellbeing, social science

Martial arts have become the center of academic attention this century. Along with groundbreaking monographs, to date, scholars have developed edited books on women in combat sports (Channon and Matthews, 2015) and on theoretical topics pertaining to habitus (Sánchez-García and Spencer, 2013). Collections have focused on the martial arts of specific regions, cultures, and intangible cultural heritage (Farrer and Whalen-Bridge, 2011; Park and Ryu, 2020). Meanwhile, special issues in journals have focused on the relationship between martial arts and society from a qualitative sociological perspective (Spencer and Hogeveen, 2014), and more recently, quantitative and biomedical perspectives on the impact of martial arts and combat sports on health (Dopico et al., 2022).

However, how martial activities might be health giving, dangerous or healing, therapeutic and rehabilitative, and how they connect with specific ideas on body and medicine remain underexplored. This Research Topic includes articles aiming at addressing themes such as revised mind-body relationships, the resurgence of mass media health messages, and the revival of specific knowledge on health and healing.

*Os Joelhos! Os Joelhos! Protective Embodiment and Occasional Injury in Capoeira*, authored by Delamont et al., draws on a long-term ethnography and 32 open-ended interviews. The paper analyses the experiences of advanced capoeira students in Britain. The students' narratives show that being a *capoeirista* means having acquired a range of strategies to prevent knee injuries as well as to adopt therapies—orthodox and alternative—necessary to ameliorate physical conditions. Significantly, this tacit shared skill is a fundamental aspect of capoeira embodiment, which previous studies on this martial activity have neglected.

*Sport Karate and the Pursuit of Wellness*, authored by Turelli et al., presents a participant-observation study about *karateka's* representation of wellness. According to the analysis, five themes are elaborated, namely, fitness, aesthetics of combat, embracing fear, aggressiveness as life posture, superiority given by control. The findings leads the authors to identify the achievement of wellness by excitement, tiredness, euphoria and body punishment. This raises important reflections regarding why and how (competitive) martial groups find wellness overflowing and where to draw the line between wellbeing and masochism.

*Blending Martial Arts and Yoga for Health*, authored by Di Placido, presents a multimodal ethnography into the Italian style of Odaka; a commodified activity which combines postural yoga with Japanese martial arts training. By considering the Odaka founders' trajectory, Di Placido argues that their background in yoga and combat sports strongly influences how health is conceived. In Odaka, the biomechanical understanding of the body, inspired by Western medical gaze, intertwines with the subtle body model of Asian traditions. Meaningfully, this interpretation shows that the commodified martial activity of Odaka does not merely expresses the self-centered ethos characterizing contemporary neoliberal society.

*Cultivating Health in Martial Arts and Combat Sports Pedagogies*, authored by Pedrini and Jennings, presents a framework to address the question “how might martial arts and combat sports be good/bad for health?” The authors conceptualize health pedagogies moving beyond a biomedical paradigm to a broader one focused on subjectivity. The Foucauldian concept of “the care of the self” helps to develop four form of “cultivation” (“self,” “shared,” “social,” “ecological”). The paper ends with methodological consideration to foster inquiries on what and how martial arts could improve personal and collective health.

In *Evidence-Based Medicine and the Potential for Inclusion of Non-Biomedical Health Systems*, authored by Langweiler, Taijiquan martial art is presented as a popular case for considering alternative medical epistemologies. The author examines the rise of Western evidence-based medicine (EBM) and contrasts it with Taoist views of evidence. EBM research protocols, with limiting timeframe and small number of movements, seems to ignore the potential beneficial properties found in Taijiquan with both long-term practice. In conclusion, Langweiler highlights the importance to face tensions between the safe public use of Taijiquan and the government accreditation to improve and ameliorate their use.

*Multilevel Evaluation of Rapid Weight Loss in Wrestling and Taekwondo*, authored by Castor-Praga et al., examines rapid weight loss (RWL) in wrestling and Taekwondo in Mexico through a cross-sectional survey. The survey reveals that 96% of the respondents use RWL, losing more than 5% of their body mass without improving sporting performances. Findings also show that the greater the relative weight loss, the greater the presence of physiological, psychological and emotional symptoms. Coaches, nutritionist and parents are those ones who play a crucial role in influencing the adoption of RWL. This call for a re-design and implementation of socio-educational interventions for preventing RWL.

“*I don't Teach Violence, I Teach Self-Control*,” authored by Domaneschi and Ricci, analyzes media representations of the MMA “right” after the murder of a boy by a gang of young men, two of whom frequented a MMA gym in center Italy. The authors focus on the most popular Italian newspaper and the Facebook group “UFC Italia.” Interestingly, MMA is not represented as an uncivilized and anti-social activity on traditional media. At the same time, traces of a “medicalization frame” appears only on UFC group. Particularly, the “healthy” features of MMA seem to rely on the coaches' ability to move the boys away from the dimension of physical danger and lack of mental self-control.

Finally, *Book Review: Martial Arts and Well-Being*, authored by Contiero, reviews one of the first academic texts on the topic by Fuller and Lloyd (2020), who offered a mixed method design using a questionnaire and in-depth interviews with martial arts instructors and athletes. The book discusses the ability of martial arts to improve balance, cognitive functions, quality of life, psychological health, and community belonging. Contiero's review proposes relevant critiques. Above everything, the exclusive attention given to some eastern martial arts raises a series of meaningful interrogatives regarding the definition of martial arts, as well as the medical philosophies and spirituality nexus underlying many martial activities. We hope that future inquires will address these and further questions.

## Author contributions

GJ wrote the first draft as lead editor of the project. LP offered comments and corrections to form a second draft while XM provided a perspective as a critical friend for the project. All authors contributed to editing and reviewing articles for the special issue as well as seeking authors and reviewers.

## Conflict of interest

The authors declare that the research was conducted in the absence of any commercial or financial relationships that could be construed as a potential conflict of interest.

## Publisher's note

All claims expressed in this article are solely those of the authors and do not necessarily represent those of their affiliated organizations, or those of the publisher, the editors and the reviewers. Any product that may be evaluated in this article, or claim that may be made by its manufacturer, is not guaranteed or endorsed by the publisher.
